# Somatostatin-IRES-Cre Mice: Between Knockout and Wild-Type?

**DOI:** 10.3389/fendo.2017.00131

**Published:** 2017-06-19

**Authors:** Cécile Viollet, Axelle Simon, Virginie Tolle, Alexandra Labarthe, Dominique Grouselle, Yann Loe-Mie, Michel Simonneau, Guillaume Martel, Jacques Epelbaum

**Affiliations:** ^1^INSERM U894, Centre de Psychiatrie et Neurosciences, Université Paris-Descartes, Sorbonne Paris-Cité, Paris, France; ^2^Laboratoire Aimé Cotton, CNRS, Université Paris-Sud, ENS Paris-Saclay, Université Paris-Saclay, Orsay, France; ^3^MECADEV UMR 7179 CNRS, Muséum National d’Histoire Naturelle, Brunoy, France

**Keywords:** cre mice, corticosterone, growth hormone secretory patterns, hepatic expression, feminization

## Abstract

The neuropeptide somatostatin (SOM) is widely expressed in rodent brain and somatostatin-IRES-Cre (SOM-cre) mouse strains are increasingly used to unravel the physiology of SOM-containing neurons. However, while knock-in targeting strategy greatly improves Cre-Lox system accuracy, recent reports have shown that genomic insertion of Cre construct *per se* can markedly affect physiological function. We show that Cre transgene insertion into the 3′UTR of the somatostatin gene leads to the selective and massive depletion of endogenous SOM in all tested brain regions. It also strongly impacts SOM-related neuroendocrine responses in a similar manner to what has been reported for SST KO mice: increased corticosterone levels after 30-min restraint stress, decreased amplitude and regularity of ultradian growth hormone secretory patterns accompanied by changes in sexually dimorphic liver gene expression (*serpina1, Cyp2b9, Cyp2a4, Cyp2d9, and Cyp7b1*). In addition to demonstrating the need for examination of the consequences of Cre transgenesis, these results also reveal how this SOM-cre strain may be a useful tool in studying the functional consequences of moderate to low SOM levels as reported in neurological and psychiatric disorders.

## Introduction

The neuropeptide somatostatin (SOM, encoded by the *sst* gene) is largely expressed in mouse brain (1). In many regions, such as the cortex or hippocampus, it is co-expressed with gamma-aminobutyric acid (GABA) and calretinin with the noticeable exception of the olfactory bulb where one-half of SOM-imunoreactive (SOM-ir) GABAergic neurons are parvalbumin-ir (PV-ir) (2). Transgenic somatostatin-Cre (SOM-Cre) mouse strains are increasingly used to unravel the physiological roles of somatostatin-containing neurons in the brain (3, 4). However, while the knock-in targeting strategy greatly improved Cre-Lox system accuracy, genomic insertion of Cre constructs *per se* can markedly affect physiological function, depending on the genomic organization of the targeted gene. In SOM-IRES-Cre mouse line [Sst^tm2.1(cre)Zjh^/J mice (4)], the Cre construct was targeted to the 3′UTR region of the *sst* gene. In this study, we characterized the molecular and physiological impact of Cre transgene insertion on the endogenous SOM tone and SOM-related responses and show an allele-dependent impairment. The massive SOM loss observed in homozygous SOM-Cre mice was associated with exacerbated corticosterone responses, feminized growth hormone (GH) secretion, and GH-dependent hepatic gene expression profiles similar to previous reports for SST KO mice. The demonstration that SOM peptide levels and SOM-related physiological responses can be strongly impacted in homozygous SOM-Cre mice needs to be taken into account when using this SOM-Cre strain.

## Materials and Methods

### Mice

SOM-IRES-Cre mice [or SST-IRES-Cre, Sst^tm2.1(cre)Zjh^/J strain, # SN13044, Jackson Lab, Bar Harbor] and SST knockout mice (5) were bred in our animal facility on a 12 h light/dark cycle with *ad libitum* access to food and water. Three- to five-month-old wild-type (WT), heterozygous, and homozygous transgenic littermates were obtained from heterozygous breeding pairs. All procedures were approved by a local ethics committee (French MESR Authorization No. 00618.04) in accordance with the European Communities Council Directive (86/609/EU).

### Quantitative RT-PCR

Freshly dissected brain regions and liver samples from SOM-Cre or SST KO mice were frozen and kept at −80°C until use. Each sample was harvested in Trizol buffer (Trizol, Life Technologies) using cold Tissue Lyzer (Qiagen) and RNA were extracted using classical phenol–chloroform protocol. 500 ng total RNA was reverse transcribed using the High Capacity cDNA Reverse Transcription Kit (Applied, Life Technologies). Real-time quantitative PCR was performed with 10 ng cDNA using an automatized 1536-well qPCR plate preparation program (BRAVO, Agilent) coupled to LightCycler PCR System (Roche, Basel), allowing simultaneous amplification for several target genes in triplicate samples. Taqman commercial probes (Life Technologies) were used for each gene [somatostatin, *sst*: Mm00436671; somatostatin receptor 1, *sstr1*: Mm00436679; *sstr2*: Mm03015782; *sstr3*, Mm00436695; *sstr4*: Mm00436710; *sstr5*, Mm01307775; Neuropeptide Y (NPY): Mm00445771; cortistatin (CST): Mm00432631; Adenylate cyclase 3, *Adcy3*: Mm00460371, Calbindin, *calb1*: Mm00486647, Calretinin, *calb2*: Mm00801461, Parvalbumin, *pvalb*: Mm00443100, insulin growth factor, *igf1*: Mm00439560, Cytochrome P450 family 2 subfamily b polypeptide 9, *Cyp2b9*: Mm00657910; *Cyp2d9*: Mm00651731; *Cyp7b1*: Mm00484157; *Cyp2a4*: Mm00487248, corticosteroid-binding globulin, *serpina6*: Mm00432327; glyceraldehyde-3-phosphate deshydrogenase, *gapdh*: Rn099999916]. mRNA expression levels were calculated according to the comparative CT method, normalized to GAPDH as reference gene, and analyzed using ANOVA statistical tests with Bonferroni *post hoc* test (StatView, SAS) (6).

### Biochemical Assays

Mouse brain samples were homogenized by sonication in 2M acetic acid. After centrifugation, supernatants were lyophilized and stored at −80°C until use. SOM levels were determined by radioimmunoassay as previously described [sensitivity threshold 0.5 pg/tube (7)], or using a commercial EIA (Phoenix Pharmaceuticals). NPY levels were determined using a commercial RIA kit (Phoenix Pharmaceuticals). Intra- and inter-assay coefficients of variation were below 10%. Data were analyzed using ANOVA with Bonferroni *post hoc* test (Statview).

### Immunohistochemistry

Deeply anesthetized SOM-Cre mice (WT and Tg/Tg littermates) were transcardially perfused with 4% paraformaldehyde (PFA). The brain was quickly removed, post-fixed 1 h in 4% PFA, cryoprotected, and then frozen in cold isopentane. 40 μm-thick coronal sections were cut with a freezing cryotome (Leica). Sections were rinsed in tris-buffered saline (TBS) and incubated in 10% normal donkey serum/0.3% Triton X100 TBS/0.05% sodium azide for 30 min, then incubated for 48 h in the same buffer with goat anti-SOM primary antibody (# D20, Santa Cruz Biotechnology, 1/1000). After several TBS rinses, sections were incubated for 2 h with A488-conjugated donkey anti-goat antibody (Invitrogen, 1/500 in TBS). After rinsing, sections were mounted on slides and coverslipped with fluoromount. Images from brain structures of WT and Tg/Tg mice were obtained under fluorescent illumination with identical settings on an AxioPlan microscope (Leica) equipped with MetaView imaging software.

### Acute Restraint Stress Response

Isolated male mice were submitted to a 30 min restraint stress in a 50 ml Falcon tube with a ventilation hole. 4 µl of blood was sampled from the tail immediately before (basal) or 30 min after stress (stressed). Corticosterone (CORT) blood levels were assayed using RIA kits (Phoenix Pharmaceuticals). Data were analyzed using ANOVA with Bonferroni *post hoc* test and Wilcoxon non-parametric test (Statview).

### Assessment of Pulsatile GH Secretion

In order to minimize stress, mice were acclimated to handling and blood sample collection for at least 2 weeks prior to the assessment of GH release. Ultradian variations of GH secretion were assessed as previously described (8, 9). Briefly, sequential tail-tip blood samples (2 μl/sample from the same tail wound for the duration of the procedure) were collected every 10 min over a 6 h sampling period (0900–1500 hours) in freely behaving animals. Blood samples were collected and homogenized in 58 µl PBS 1×-0.05% tween on 96-well plates kept on ice, then transferred to −20°C for storage. GH concentrations were determined using an in-house mouse GH ELISA as previously described (10) and plotted as a function of time (8, 9). Cumulative distributions of relative frequencies were analyzed using the two-sample Kolmogorov–Smirnov test (Prism, GraphPad Inc.).

### Behavioral Testing

Mice were individually housed a week before the tests.

#### Elevated plus-Maze

Each mouse was placed at the center of the maze (38 cm × 7 cm arms, 50 cm above the floor, 50 lux) with the head facing an open arm and the percentage of entries and time spent into open or closed arms were recorded during the 8 min-session using Videotrack Software (View Point S.A., Champagne au Mont d’Or, France).

#### Open field

Each mouse was placed in a corner of a 50 cm × 50 cm × 45 cm square open field and its activity was recorded during 20 min in the peripheral (10 cm wide) or central (30 cm wide) zones. Global activity as well as distance and time spent in the central zone were quantified using Videotrack Software.

#### Forced-Swim Test

Each mouse was placed in a beaker filled with water (23°C) for 6 min. The sessions were recorded using Videotrack Software and the time spent swimming, climbing, or staying immobile during the session was analyzed *a posteriori* by a trained experimenter unaware of genotype.

Data were analyzed using ANOVA and Bonferroni *post hoc* test, using repeated measures for open field activity (Statview).

## Results

**Figure 1:**
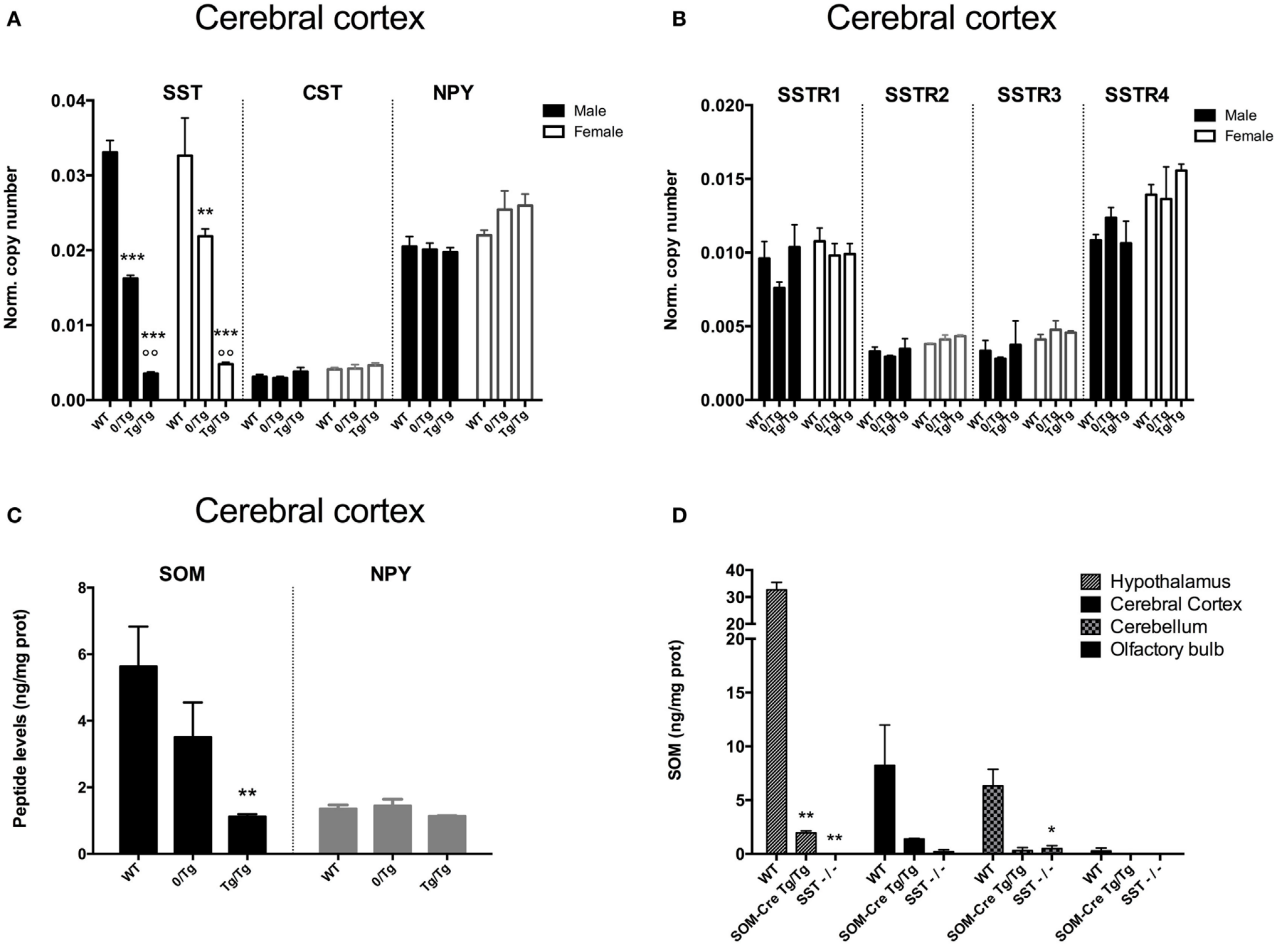
Effect of genotype on peptide and somatostatin receptor subtype levels in mouse brain. **(A)** Somatostatin (SST), cortistatin (CST), and neuropeptide Y (NPY) expression levels in wild-type (WT), heterozygous (0/Tg), or homozygous (Tg/Tg) SOM-Cre male (black bars) and female (open bars) cortex (*n* = 3–4 mice per group). Error bars indicate SEM. ***P < 0.001 vs WT, ^°^*P* < 0.01 vs 0/Tg. **(B)** SSTR1, SSTR2, SSTR3, SSTR4 expression levels in WT, heterozygous (0/Tg) or homozygous (Tg/Tg) SOM-Cre male (black bars) and female (filled bars) cortex (*n* = 3–4 mice per group). Error bars indicate SEM. **(C)** SOM and NPY peptide levels in male WT, heterozygous (0/Tg) or homozygous (Tg/Tg) SOM-Cre cortex (*n* = 6–7 mice per group). Error bars indicate SEM. **P < 0.01 vs WT. **(D)** SOM peptide levels in the hypothalamus, cortex, cerebellum, and olfactory bulb of male WT, homozygous SOM-Cre (Tg/Tg) or SST KO (SST^−/−^) mice (*n* = 2–5 mice per group). Error bars indicate SEM. *P < 0.05, **P < 0.01 vs WT.

As shown in Figure 1A, somatostatin expression (SST) was strongly reduced in the cortex of heterozygous and homozygous SOM-Cre mice for both sexes (genotype effect, *P* < 0.0001, male *P* < 0.0001, female *P* < 0.01) as compared to WT. Heterozygous (0/Tg) transgenic mice showed a 51 and 34% decrease in males and females, respectively, as compared to WT. Homozygous (Tg/Tg) transgenic mice showed an even greater decrease: 89% for males, and 86% for females. In contrast, CST, NPY (Figure 1A), and SSTR1-4 (Figure 1B) mRNA levels were not significantly different in transgenic and control animals. Decreased SST expression was also detected in the hypothalamus in SOM-Cre mice (0/Tg: 50%, males and 58%, females; Tg/Tg: 92%, males and 87%, females), the hippocampus (0/Tg: 49%, males and 36%, females; Tg/Tg: 90%, males and 91%, females), or the olfactory bulb (0/Tg: 42%, males and 48%, females; Tg/Tg: 89%, males and 100%, females), three regions with highly differing SOM expression levels in the WT mice (*data not shown*). Except SST, no major changes in expression were found for the other markers (listed in Section “Materials and Methods”) tested in heterozygous or homozygous SOM-Cre mice (data not shown).

Somatostatin concentration (SOM) was measured in the cortex of WT, 0/Tg, and Tg/Tg SOM-Cre male mice. A 30% decrease was found in 0/Tg mice and SOM levels fell to 70% of WT in Tg/Tg mice (Figure 1C) while NPY levels did not change. A massive decrease of SOM peptide levels in Tg/Tg mice was also detected in the hypothalamus, cerebellum, and olfactory bulb (70–90% decrease), while SST KO mice (SST^−/−^) were used as negative controls (SOM-Cre vs WT, *P* < 0.0001; SST KO vs WT, *P* < 0.0001; hypothalamus vs all other regions, *P* < 0.0001; Figure 1D).

**Figure 2:**
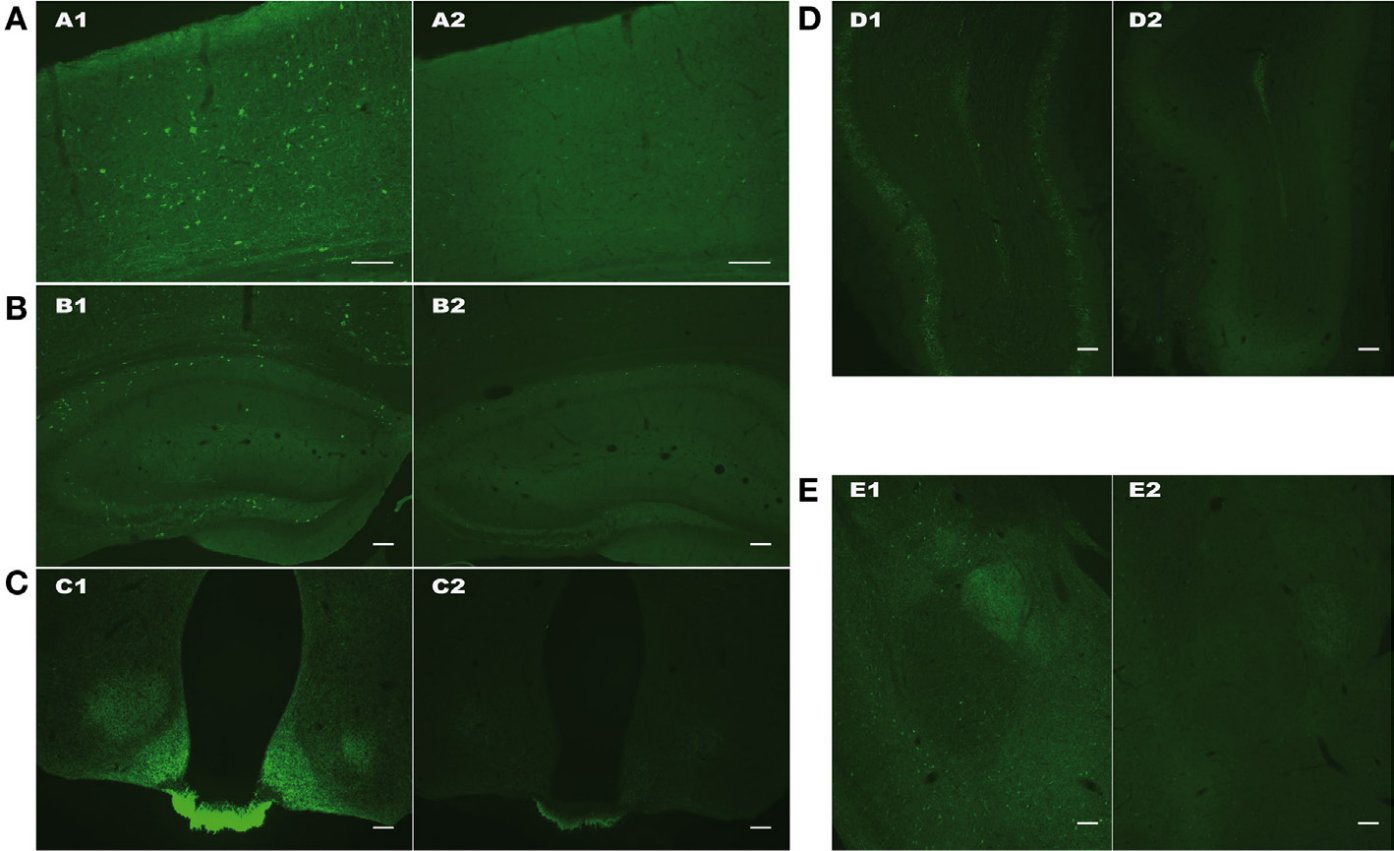
Representative pictures of SOM immunoreactivity in cortex **(A)**, hippocampus **(B)**, hypothalamus **(C)**, olfactory bulb **(D)**, and amygdala **(E)** of WT (1, left) or homozygous Tg/Tg SOM-Cre (2, right) mice. Scale bars: 100 µm.

As shown on Figure 2, both cellular and fiber somatostatin labeling was strongly affected in SOM-Cre Tg/Tg brain, in cortex, hippocampus, hypothalamus (with a striking decrease of the staining in the median eminence), olfactory bulb, and amygdala (Figures 2A–E).

**Figure 3:**
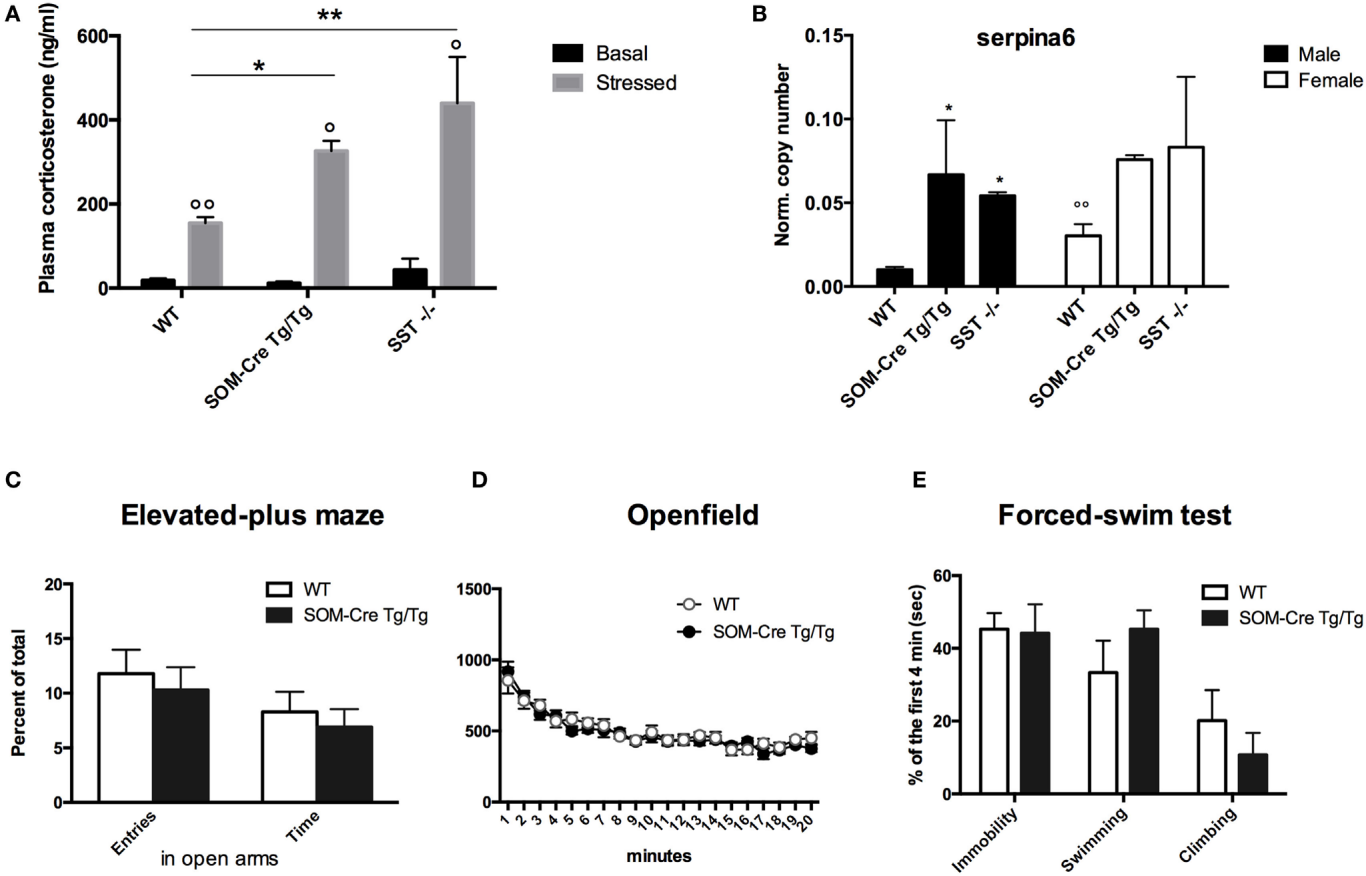
Increased stress responses in homozygous SOM-Cre and SST KO mice. **(A)** Plasma corticosterone before (black bars) or after a 30-min restraint stress (gray bars) (*n* = 5–9 per group). Error bars indicate SEM. *P < 0.05, **P < 0.01 vs WT, ANOVA; ^°^*P* < 0.05, ^°°^*P* < 0.01 vs Basal, Wilcoxon test. **(B)** *Serpina6* expression levels in wild-type mice (WT), homozygous SOM-Cre (Tg/Tg), or SST KO (SST^−/−^) male (black bars) and female (white bars) mice (*n* = 3–9 mice per group). Error bars indicate SEM. *P < 0.05 vs WT, ^°°^*P* < 0.001 vs male, ANOVA. **(C–E)** Behavioral responses of WT (white bars) and SOM-Cre Tg/Tg (gray bars) male mice. **(C)** Percent of total entries and time spent in the open arms in the elevated-plus maze. **(D)** Global activity in the open field test (20 min) **(E)** Percent of time spent in immobility, or swimming or climbing activity during the four first minutes of the forced-swim test (*n* = 10 mice per group). Error bars indicate SEM.

Since *sst* gene deletion affects both HPA axis and hormonal regulation of stress (11–13), basal and stress-induced plasma corticosterone (Cort) levels were assayed in WT, homozygous SOM-Cre, and SST KO mice. Basal Cort levels were not statistically different [*F*(2, 16) = 1.363, P ns; WT 18.2 ± 5.3 (*n* = 9), SOM-Cre Tg/Tg 11.6 ± 4.0 ng/ml (*n* = 5) KO SST 43.1 ± 26.9 ng/ml (*n* = 5)]. 30 min-restraint stress-enhanced corticosterone levels in all groups [stress effect *F*(1, 2) = 6.607, *P* < 0.01] with a higher response in SOM-Cre and SST KO mice [genotype effect: *F*(2, 16) = 8.349, *P* < 0.01, Figure 3A, genotype × stress interaction *F*(1, 2) = 6.607, *P* < 0.01 Figure 3A]. *serpina6*, a female predominant hepatic gene, which encodes corticosteroid-binding globulin, showed increased expression in male SOM-Cre Tg/Tg mice [genotype effect in males *F*(2, 17) = 6.206 *P* < 0.01, Figure 3B] (11), suggesting feminization in SOM-Cre Tg/Tg males, as already reported in SST KO mice (Figure 3B) (11). However, no major emotional or motor phenotype was detected in Tg/Tg SOM-Cre when compared to WT in the elevated plus maze, open field, or forced-swim tests (Figure 3C).

**Figure 4:**
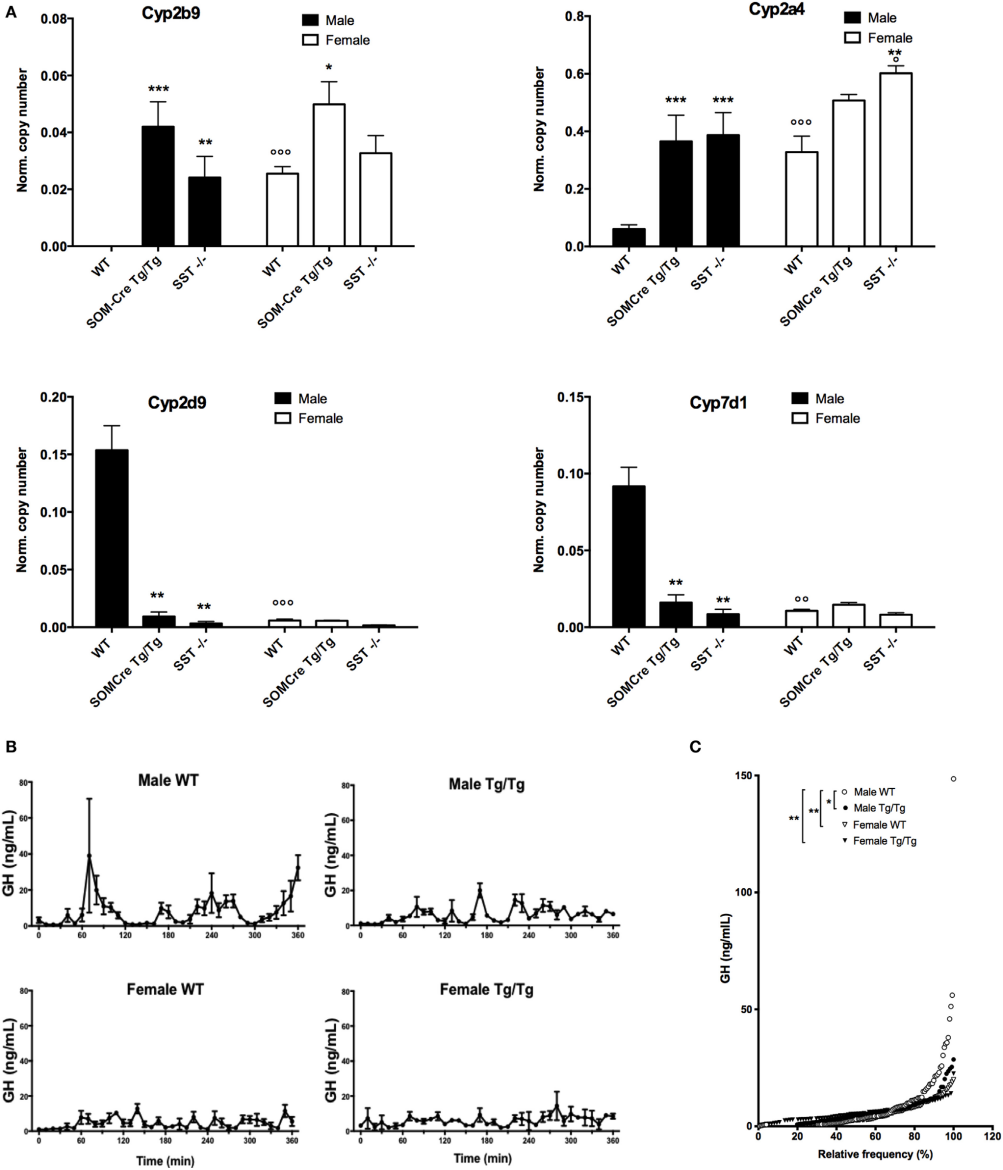
Feminized molecular and hormonal profiles of homozygous SOM-Cre and SST KO male mice. **(A)** Expression levels of female-predominant gene Cyp2b9 and Cyp2a4 and male-predominant Cyp2d9 and Cyp7d1 genes in wild-type mice (WT), homozygous SOM-Cre (Tg/Tg), or SST KO (SST^−/−^) male (black bars) and female (filled bars) mice (*n* = 3–4 mice per group). Error bars indicate SEM. **P < 0.01, ***P < 0.001 vs WT, ^°^*P* < 0.05, ^°°^*P* < 0.001 vs male, ANOVA. **(B)** Mean growth hormone (GH) profiles over 6 h sampling. **(C)** GH cumulative distribution in WT (open symbols) and SOM-Cre Tg/Tg (black symbols) males (circles) and females (triangles) (*n* = 2–4 mice per group). Error bars indicate SEM. *P < 0.05, **P < 0.01 vs WT male, Kolmogorov–Smirnov test.

Growth hormone-dependent mRNA levels for typical highly sexually dimorphic hepatic genes were then measured using RT-QPCR. As shown in Figure 4A, female-predominant (Cyp2b9, Cyp2a4) as well as male-predominant (Cyp2d6, Cyp7b1) genes exhibited robust differences in SOM-Cre Tg/Tg and SST KO as compared to WT mice, converging toward a feminized expression profile in the mutant mice (sex effect for Cyp2b9, Cyp2a4, Cyp2d9 and Cyp7b1, respectively: *P* < 0.01, *P* < 0.05, and *P* < 0.05, genotype effect: *P* < 0.0001, *P* < 0.001, and *P* < 0.05, sex × genotype effect *P* = 0.06, *P* < 0.01, *P* < 0.01).

Since sexually dimorphism is highly dependent on GH secretion, GH secretory pulses were assayed during six consecutive hours in SOM-Cre WT and Tg/Tg males and females. Figure 4B illustrates mean GH secretion profiles and shows that typical male secretory profiles are no longer observed in SOM-Cre Tg/Tg males, as previously reported in SST KO males (11). SOM-Cre Tg/Tg male profiles rather show a feminized pattern (i.e., secretory peaks are more frequent and of less amplitude), similar to the one observed in WT and Tg/Tg females (Figure 4B). Analysis of the cumulative frequency distributions (Figure 4C) showed that WT male GH distribution was significantly different from all other groups (SOM-Cre Tg/Tg male *D* = 0.3871, *P* < 0.05; female WT *D* = 0.4194, *P* < 0.01; SOM-Cre Tg/Tg female *D* = 0.4194, *P* < 0.01, Kolmogorov–Smirnov) while SOM-Cre Tg/Tg male GH distribution was not statistically different from each female group.

## Discussion

The application of Cre-based transgenic models using promoter-driven expression of Cre recombinase greatly improved our knowledge of anatomical connections and permitted the physiological dissection of cellular properties in neural circuitry (14, 15). Crossing such models with mice expressing floxed fluorescent reporter proteins or gene-regulatory sequences allows for detection, targeting of genetic addition- or inactivation of a given gene [see Ref. (16) for review]. Several pitfalls have yet emerged, calling for the careful evaluation of appropriate controls prior to drawing metabolic or physiological conclusions (14, 17). According to the spatial or temporal activation profile of the promoter used, it is recommended to carefully compare the distribution of promoter-driven Cre with the endogenous expression profile of the targeted gene. In particular, transient ontogenic expression of target genes can lead to permanent changes revealed at later stages as off-target effects (18).

The initial stage of Cre transgene insertion *per se* must also be cautiously considered when using Cre-based technology since it can impact the function of this region. The commonly used transgenic strain SST-IRES-Cre line studied here was generated by insertion of IRES-Cre sequence in the 3′UTR of the SST gene (4). Taking advantage of the IRES sequence, this strategy allows generating a bicistronic transcript able to code both SST and cre. Unexpectedly, endogenous somatostatin expression is selectively and dose-dependently disrupted in homozygous mutants and the fall in somatostatin levels affects neuroendocrine and physiological responses, mirroring the previously reported dramatic changes observed in SST KO mice (11, 13). Using UCSC Genome Browser tools, we did not evidence any conserved motives of transcription factor sites, chip-seq positive signals, or predicted miRNA signals in this part of the SST 3′UTR. The decrease in expression reported here can possibly reflect a structural sensitivity of the mouse SST gene, as already suggested for the transcriptional repressor methyl-CpG binding protein 2 (MeCP2) effect on SST gene expression (19). Thus, cautious examinations of the other SOM-Cre strains available with distinct targeting sites for transgene integration (4, 14, 20) is needed.

While SST expression is selectively impaired in SOM-Cre transgenic mice, no major changes were found concerning SSTR expression and expression levels of the typical peptides or markers assayed in the different brain structures. Except for SST, heterozygous expression profiles were always similar to WT, showing no main significant differences with homozygous mice. Molecular and biochemical approaches quantified the decrease in SOM levels that was also evidenced when comparing *in situ* hybridization data available in Allen Brain Atlas. This indeed suggests that there is reduced Cre labeling in Sst-IRES-Cre SOM cortex as compared to Sst labeling in C57Bl/6 mice at similar cortical levels [(14); http://mouse.brain-map.org].

SOM decrease *per se* correlates well with the neuroendocrine readouts previously shown in SST KO mice, either the dimorphic GH-dependent hepatic genes or the higher CORT response to restraint stress. This strongly supports the hypothesis that SOM is required for masculinization of the GH axis and behavioral emotionality control, as previously demonstrated in recent studies (11, 13, 21). Conversely, neuroendocrine function did not correlate with behavioral emotionality since no major emotional phenotype was found in the behavioral studies (Figure 3) as previously reported for SST KO males and females (11, 13, 21). No significant basal plasma CORT difference was found between WT and SOM-Cre or SST KO homozygous animals in the present study, while stress response was strongly enhanced in the SOM-Cre Tg/Tg mice, as previously reported for SST KO (11). Herein, as in Adams’ study, mice were housed individually for the behavioral and restraint test, a condition that favors the emergence of stress susceptibility. This may explain the differences observed with Lin and Sibille study (21) where basal levels are different and response to acute stress is similar to WT levels. Nonetheless, increased expression in SOM-Cre Tg/Tg males of the *serpina6* gene, coding the corticosterone binding protein, which traps major circulating corticosterone at the periphery, mirrors plasma CORT increase as previously reported in SST KO mice (11, 13).

In conclusion, inserting *cre transgene* in *sst* 3′UTR allele-dependently knocks down endogenous SOM expression in the Sst^tm2.1(cre)Zjh^/J mice. Therefore, such limitations should be taken into account for conditional KO (cre-lox) studies. On the contrary, these mice may serendipitously constitute an interesting model to study the functional consequences of the gradual changes of somatostatin, which take place (i) transiently during brain development [see Ref. (22) for review] and (ii) in an irreversible manner in major neurological and psychiatric diseases such as Alzheimer’s disease, major depression, and schizophrenia (1, 21, 23, 24).

## Ethics Statement

All procedures were approved by a local ethics committee (French MESR Authorization No. 00618.04) in accordance with the European Communities Council Directive (86/609/EU).

## Author Contributions

CV and JE designed and supervised the study and they wrote the manuscript, AS performed the immunohistochemical experiments, DG the biochemical assays, VT and AL performed the GH study, GM the Cort experiments, and YL-M and MS bioinformatic survey of the SST promoter. CV performed molecular biology and behavioral experiments, analyzed all data, and prepared the figures.

## Conflict of Interest Statement

The authors declare that the research was conducted in the absence of any commercial or financial relationships that could be construed as a potential conflict of interest. The reviewer, PP, and handling editor declared their shared affiliation, and the handling editor states that the process nevertheless met the standards of a fair and objective review.

## Abbreviations

IRES, internal ribosomal entry site; KO, knockout, SOM, SST, somatostatin; SSTR, somatostatin receptor.
